# Control mechanisms for stochastic biochemical systems via computation of reachable sets

**DOI:** 10.1098/rsos.160790

**Published:** 2017-08-23

**Authors:** Eszter Lakatos, Michael P. H. Stumpf

**Affiliations:** Centre for Integrative Systems Biology and Bioinformatics, Department of Life Sciences, Biosciences, Imperial College London, London SW7 2AZ, UK

**Keywords:** molecular noise, synthetic biology, reachability analysis, stochastic control, model invalidation

## Abstract

Controlling the behaviour of cells by rationally guiding molecular processes is an overarching aim of much of synthetic biology. Molecular processes, however, are notoriously noisy and frequently nonlinear. We present an approach to studying the impact of control measures on motifs of molecular interactions that addresses the problems faced in many biological systems: stochasticity, parameter uncertainty and nonlinearity. We show that our reachability analysis formalism can describe the potential behaviour of biological (naturally evolved as well as engineered) systems, and provides a set of bounds on their dynamics at the level of population statistics: for example, we can obtain the possible ranges of means and variances of mRNA and protein expression levels, even in the presence of uncertainty about model parameters.

## 1. Introduction

Much of the research in synthetic and systems biology in the last decade has focused on the study of elementary biological systems. This has often been with the aim of controlling and modifying them in order to achieve new functional modules exhibiting novel and useful behaviour [1], such as sustained oscillations [2] and bistability [3]. It is now becoming possible to use engineered biological systems made of characterized components to solve specific problems, such as information processing, energy production or production of chemicals.

However, biological systems are inherently noisy and probabilistic in nature, which can pose significant difficulty for one aiming for a reliable, well-characterized module. Although several external control techniques have been developed which are able to avoid some of the variability in a population [4,5], noise at the level of molecular processes is often unavoidable and does, for example, affect quite profoundly how information is transmitted along the molecular networks underlying cell function [6–8]. Furthermore, due to the unreliability of measured quantities, our understanding of the underlying mechanisms might be mistaken leading to sub-optimal analysis and design. Therefore, we have to assess the practical limits on the amount of noise in a general biological module, in order to evaluate the efficiency of a control design or the reliability of a mechanistic model.

Reachability analysis has been widely used in control design and engineering for applications such as verification of electrical or mechanical networks and hybrid automata [9–13]. The analysis focuses on the computation of the subset of the state-space that can be reached within a certain time-limit, given some starting position of the system and external inputs. The technique can also be used to verify that a certain undesired state is not reached under realistic operating condition, or that the behaviour of the system is robust and qualitatively/quantitatively holds for different conditions, including different realistic inputs to the system.

To this end, reachability is generally calculated under varying levels of uncertainty regarding details of the system—such as initial state, input signal and rate parameters—usually formalized by assuming that these parameters come from a set of plausible values. Therefore, unless analytical solutions are derived for some abstraction of the system, the applicability of the computation heavily relies on the choice of the set representation [11,14,15]. Some representations might prove computationally expensive and hence impractical for high-dimensional systems, while a simple shape representation can lead to crude over-approximation of the reachable set. Methods have been proposed using several techniques such as polygonal projections [16], oriented hyper-rectangles, special polyhedra [17], ellipsoids [15] or level sets [11]. In this work, we use zonotopes [9], a centrally symmetric type of polytopes that can be conveniently represented by a list of vectors.

Although there is already a substantial body of work on reachable set computation for problems in engineering, including highly nonlinear cases [18], hybrid automata [19] and differential-algebraic equations [20], the complexity, frequent nonlinear behaviour and strict constraints on many of the model parameters of biochemical systems require the development of specialized analysis methods. There are already a few applications of reachability techniques to biological examples with special emphasis on the treatment of the nonlinearity of the system, either through direct computation [21] or through hybridization-based methods using either static or dynamic partitioning of the state-space [22–24]. The work by Dang *et al.* [24] has also been expanded to take into account the possible lack of knowledge of parameter values [25]. The stochasticity in biological systems has been tackled in even more diverse ways: through computing bounds on the probability function [26], using stochastic hybrid systems [27], or analytically deriving invariant sets for linear equations obtained from the stochastic model [28].

In this work, we propose a computationally efficient and flexible method to compute the reachable set (in a zonotopic representation) of stochastic biochemical systems; besides stochasticity we also consider possibly nonlinear rate laws; controlled or uncertain input signals; and uncertainty about model parameters, including those which we may need to control. The main steps behind our derivation are the following: (i) we first obtain an ordinary differential equation (ODE) representation of the system’s mean and (co-)variances; (ii) then use an iterative procedure to obtain a conservative approximation of consecutive reachable sets up to a final time of interest. Here, we primarily use the linear noise approximation (LNA) for the first step, but also consider the moment expansion approximation [29–31] to demonstrate that other methods with different applicability can be used equally well in order to generate equations for the second step. We derive a new method to tightly approximate realistic biological input signals, and piece-wise temporal linearization is employed to deal with common nonlinearities. We also give conservative approximation formulae for the reachable set when rate parameters of the system are not precisely known—which is very often the case in biochemical systems [32].

The method is first demonstrated on two elementary modules fundamental to mathematical models and regulatory designs of biochemical networks. The first system presents the use of reachability analysis for the study of noisy biochemical reactions and evaluating a control on the levels of cell heterogeneity; the second example considers the task of model (in)validation for cases when high cell-to-cell variability poses a challenge to estimating the system’s true behaviour. Finally, we explore the limitations of our method and how it can be used as a quick indicator of ‘challenging’ dynamics.

## 2. Material and methods

### 2.1. Zonotopes

A zonotope [33] is described by the position of its centre (*c*) and a set of generator vectors (*g*_1_,…,*g*_*p*_) as

2.1 $$Z:=\left\{x\in R^{n}\;|\;x=c+\sum_{i=1}^{p}a_{i}g_{i},\;-1\leq a_{i}\leq 1\right\}.$$

Two example zonotopes are shown in the electronic supplementary material, figure S1(a). In the following, we use the shortened notation (*c*;*G*) to represent Z, where $G\in R^{n\times p}$ is a generator matrix formed from the generator column vectors. Zonotopes are a convenient representation as they are closed under Minkowski-addition and affine transformations, the two key operations in reachability analysis [9]. Furthermore, the above can be calculated through simple matrix-vector operations: given zonotopes Z=(c;G) and $W=(d;H),\;(H\in R^{n\times q})$ and the affine transformation T(x)=Ax+b,

2.2*a* Z⊕W={x+y | x∈Z, y∈W}=(c+d;[G,H])

and

2.2*b* T(Z)={Ax+b | x∈Z}=(Ac+b;AG),

where [*G*,*H*] is the concatenation of the ‘vector lists’.

### 2.2. Linear noise approximation

Given a stochastic system with *N*-dimensional state variable *χ*(*t*) describing the abundance of modelled species at time *t*. We assume that the system is defined by (i) a stoichiometry matrix, *S*, in which each element (*S*_*ij*_) describes the amount of *i* molecules gained through reaction *j*; and (ii) a collection of reaction propensities, *F*=[*a*_1_, *a*_2_,…,*a*_*r*_]^T^ that quantifies the probability of each reaction happening, similarly to reaction speeds in classical deterministic systems. A detailed illustration of the stochastic formalism is given in the first example in §4.

In the LNA [34], we divide the state variable into a macroscopic part, and random fluctuations, as *χ*(*t*)=*ϕ*(*t*)+*ξ*(*t*). This way the original system, which is typically described by the time-evolution of the entire probability distribution [35], is converted into an ordinary and a stochastic differential equation (in the Ito form),

2.3 $$\begin{array}{rl} \frac{d\phi (t)}{dt} & =S\cdot F(\phi (t))\\ \;\text{and}\;\frac{d\xi (t)}{dt} & =S\cdot D(\phi (t))\xi (t)+\sum_{j=1}^{r}S_{:,j}W_{j}(a_{j}(\phi (t))), \end{array}\}$$

where *W*_*j*_(*a*_*j*_(*ϕ*(*t*))) are independent Wiener processes (∀*j*) with normally distributed increments, $N(0,a_{j}(\phi (t)))$; and {*D*(*ϕ*)}_*ik*_=∂*a*_*j*_(*ϕ*)/∂*ϕ*_*k*_, hence *D*=∂*F*/∂*ϕ* is the Jacobian of the system.

In the applications we aim to investigate, instead of trajectories of individual realization (i.e. single cells), we are interested in the population-level behaviour. Therefore, equation (2.3) is used to obtain equations that describe how the mean and variance of the probability distribution of values of *χ*(*t*) changes. The decomposition of the state variable makes it straightforward to follow the change in the mean of the system, as the macroscopic part corresponds to the mean behaviour and hence the evolution it is already given by equation (2.3). The evolution of the covariance matrix ($\Sigma \in R^{N\times N}$) is calculated as

2.4 $$\frac{d\Sigma }{dt}=SD\Sigma +\Sigma {\left(SD\right)}^{T}+S\cdot \text{diag}\{F(\phi )\}\cdot S^{T},$$

where diag{*F*} represents the *r*×*r* matrix, whose diagonal elements are those of *F* (i.e. diag{*F*}_*ii*_=*F*_*i*_) and all other elements are zero.

Thus a set of ordinary differential equations can be derived that follows the time-dependent change of the mean values and (co)variances of all species, summarized in the $x(t)\in R^{n}$ vector with *n*=*N*(*N*+3)/2 elements. We obtain *x*(*t*) by concatenating *ϕ*(*t*) and the vectorized form of the upper triangle of *Σ*(*t*) denoted as (*Σ*(*t*))^*v*^,

$$\frac{dx}{dt}=\frac{d}{dt}\left[\begin{array}{c} \phi (t)\\ {\left(\Sigma (t)\right)}^{v} \end{array}\right]=\left[\begin{array}{c} S\cdot F(\phi (t))\\ {\left(SD\Sigma (t)\right)}^{v}+{\left(\Sigma (t){\left(SD\right)}^{T}\right)}^{v}+{\left(S\;\text{diag}\{F(\phi (t))\}S^{T}\right)}^{v} \end{array}\right]=f(x(t)),$$

where the upper triangle vectorization operator stands for row-continuous listing of all elements in and above the diagonal: for example (*Σ*(*t*))^*v*^=[*Σ*_11_,*Σ*_12_,…,*Σ*_1*N*_,*Σ*_22_,…,*Σ*_2*N*_,*Σ*_33_,…,*Σ*_*NN*_]. We give an *N*=2 dimensional example of this computation in the first example of §4.

### 2.3. Moment expansion approximation

The LNA is based on the assumption that the system noise is well described by a normal distribution, and molecules are present in at least moderately high amounts. In many cases, it offers a good approximation even when these conditions are not met. However, to handle more problematic cases, we also use moment expansion approximation [29,30], a less constrained moment generating method, to obtain ordinary differential equations.

In brief, given the aforementioned stochastic system formed by *χ*(*t*), *S* and *F*, one can use the moment generating function of the species’ probability distribution to obtain expressions of the time-evolution of *k*th order moments. This can be derived and evaluated to any arbitrary value of *k*. However, for nonlinear systems every moment equation would depend on moments of subsequent orders, leading to an infinite set of equations. Therefore, we also need to apply moment closure formulae (mc^*k*^) to substitute the highest order terms ($E(\chi^{k})$) with expressions of means (*ϕ*(*t*)) and (co)variances (*Σ*(*t*)): $E(\chi^{k})⟵{\mathrm{mc}}^{k}(\phi (t),\Sigma (t))$.

$$E(\chi^{k})⟵{\mathrm{mc}}^{k}(\phi (t),\Sigma (t)).$$

A major difference between applying the LNA or moment expansion techniques is that the latter can be applied to any arbitrary order or moments, such as skewness, kurtosis, etc. However, for reachability analysis our primary interest is in the mean and variance of molecules of the system, and moment expansion can be used in three ways to derive these. We can (i) set the expansion to be only up to second-order moments; (ii) use the step in the above equation to substitute all higher-order moments, not just the highest order ones; or (iii) use the whole system of *k* moments as our state vector, to make sure no essential influence on means and variances is omitted.

There has been considerable discussion of moment equations and their applicability to the analysis of stochastic systems, see e.g. [36–38]. We, therefore, refer to [29,30] for the applicability and limitations of this particular method and mathematical details of the derivation. Furthermore, we suggest the use of the Python-based implementation, MEANS [31], for automatic computation of the step converting the stochastic system into a deterministic ODE. The package provides a user-friendly framework that only requires the stochastic description to output the desired equation set, and offers both the linear noise and the moment expansion approximation for equation generation.

### 2.4. State-space representation

Next we consider the representation of the deterministic system obtained via either of the aforementioned approximation techniques. The general system is described by the evolution of the *n*-dimensional state variable, *x*(*t*) as in [39]

2.5 $$\dot{x}(t)=f(x(t),u(t));\;x(0)=x_{0},$$

where $u(t)\in R^{m}$ is an input signal, and *f*(*x*,*u*) is a generally nonlinear but time-independent transition function. We focus on the case where the input-dependence in the above equation can be separated as

f(x,u)=g(x)+Bu,

where $B\in R^{n\times m}$ is the input matrix, which specifies which states are affected by the inputs. In terms of the original stochastic system, *u* can be understood, for example, as a zeroth-order (constitutive production) reaction, as we will discuss through the first example in §4.

We further assume that instead of *x*_0_, a set containing all possible initial values in the state-space, I, is given—for example, because we observe a range of expression values in a population of cells. Similarly, the input signal may also come from a set, U, which is bound by some value μ∈R so that ∥*u*(*t*)∥≤*μ* ∀*t*. Then equation (2.5) is presented as a differential inclusion [40] of the form

2.6 $$\dot{x}(t)\in g(x(t))⊕BU;\;x(0)\in I.$$

The reachable set of this system at any time *t* is defined as

2.7 $$R_{t}:=\{y\in R^{n}\;|\;\exists (x,u);\;x(0)\in I,\;u(s)\in U,\;x(s)\text{ follows }\left(\text{2.6}\right)\;\forall s\leq t,\;y=x(t)\},$$

i.e. all the states we can achieve by having the above described system start from a possible initial state and affected by plausible input signals. Similarly, we can define $R_{\left[0,t\right]}$ as all the states reachable within the time-interval [0,*t*], by computing all individual reach sets: $R_{\left[0,t\right]}=\underset{s\in [0,t]}{⋃}R_{s}$.

## 3. Reachable set computation

We start our derivation by considering the case when *f* is a linear function of *x*; under such condition the system can be represented in the linear time-invariant (LTI) form,

3.1 $$\dot{x}(t)=Ax(t)+Bu(t);\;x(0)=x_{0},$$

where $A\in R^{n\times n}$ is the state (transition) matrix. We focus on single-input systems, where *B* is an *n*×1 matrix (i.e. a column vector); however, all results can be easily generalized to other values of input dimension, *m*. The solution of this system is generally given as

3.2 $$x(t)=e^{At}x_{0}+\int_{0}^{t}e^{As}Bu(t-s)\;ds,$$

which can be further simplified through assuming a constant input signal and an invertible transition matrix

3.3 $$\int_{0}^{t}e^{As}\;ds\cdot Bu=A^{-1}(e^{At}-I)Bu,$$

where *I* is the *n*×*n* identity matrix. In the following for convenience, we use the notation

$$\psi (A,t)=A^{-1}(e^{At}-I).$$

We consider an equidistant partitioning of the time horizon into *N* intervals, with time-step *τ*=*T*/*N*. We start the derivation of reachable sets from I to $R_{T}$ by handling the case of the autonomous system, i.e. when *u*≡0.

Given a reachable set at an arbitrary time-point, $R_{t}=(c;G)$, the new set reached under zero input can be calculated according to equation (3.2), as

3.4 $$R_{t+\tau }=e^{A\tau }R_{t},$$

since the second term becomes zero for autonomous systems. This operation is in fact an affine transformation x→Mx (with *M*=e^*Aτ*^) and hence the resulting set will also be a zonotope, as given by equation (2.2b),

$$R_{t+\tau }=(e^{A\tau }c;\;e^{A\tau }G).$$

As the same equation applies for all values of *t*, the transition matrix can be applied iteratively to propagate I up to the final reach set, $R_{T}$. If the aim is to map the entire space the system explores between times 0 and *T*, the reachable sets of the time-intervals, $R_{\left[i\tau ,(i+1)\tau \right]}$ has to be derived from the two end-points. Generally, this can be done by computing the convex hull of $R_{i\tau }$ and $R_{(i+1)\tau }$ and then extending this set to contain all affine solutions for one time-step (see [14]; electronic supplementary material, figure S1(b) for an illustration). However, zonotopes are not closed under this operation and it is desirable to avoid the exact computation of a convex hull. We use the conservative (over-)approximation presented in [9] to derive a set enclosing all points reachable between 0 and *τ*. All following intervals can be propagated from this set, as for all *t*∈[0,*τ*], $R_{t}\in R_{\left[0,\tau \right]}$ and hence $e^{A\tau }R_{t}\in e^{A\tau }R_{\left[0,\tau \right]}$.

The above computation is easily extended to account for a constant input signal, *u*. According to equations (3.3) and (3.4), $R_{t+\tau }$ is given by

$$R_{t+\tau }=e^{A\tau }R_{t}+A^{-1}(e^{A\tau }-I)Bu,$$

which is conveniently also a zonotope, as *A*^−1^(e^*Aτ*^−*I*)*Bu*=*ψ*(*A*,*τ*) is a constant vector. However, in systems with uncertainty (so, for example, where the input signal is unknown) we need to transform and enlarge, or *bloat*, the set $R_{i\tau }$ in each iteration, to account for the system dynamics under all admissible input/parameter values. This corresponds to taking all combinations of an unknown initial value (contained in I) and unknown parameter (input signal or reaction rate value) and obtaining every state the system can thus possibly reach. This is generally done by taking the Minkowski sum of the reachable set propagated from I, $R_{t+\tau }$; and the ‘bloating’ sets, *β*_*μ*_ and *β*_*δ*_, accounting for the uncertainty introduced in one time-step by the input or rate parameter set, respectively. In the following sections, we derive these bloating sets in a zonotope formalism for convex, one-dimensional sets of input and parameter values. In addition, see electronic supplementary material, figure S2 for demonstration of the computational steps.

To summarize, using the short form $R_{i}$ to denote either $R_{i\tau }$ or $R_{\left[(i-1)\tau ,i\tau \right]}$ given in a zonotope form, the next reachable set is calculated as

3.5 $$R_{i+1}=(e^{A\tau }c_{i};e^{A\tau }G_{i})⊕\beta_{\mu }⊕\beta_{\delta }.$$

As zonotopes can be stored as a set of column vectors summarized in a matrix, all the above computational steps are typically executed very efficiently, especially in languages dedicated for matrix-vector operations. Additionally, functions of the transition matrix and the constant time-step—such as e^*Aτ*^—have to be calculated only once through the initialization of the algorithm, providing a further gain in computational speed.

### 3.1. Input for biological systems

In the algorithm proposed by Girard [9] the input set, U is taken to be an *n*-dimensional hypercube enclosing all points between [−*μ*,*μ*] in all dimensions: equivalent to the radius *μ* ball in the infinity norm. Such a generalized approximation of the input set is sometimes necessary, as the analysis is motivated by determining either a target or an avoidable set in the state-space, and the input set is meant to capture all variations induced by noise in a physical system.

In the case of biological inputs, however, we usually have more detailed information about U, meaning that the aforementioned general input set would contain many implausible signals, and hence the over-approximation would be too loose to provide useful information on the actual reachable sets. For example, in the typical case the control input is implemented as a certain type of molecular species added to the system; this cannot take negative values and it is also reasonable to assume an upper limit on the number of molecules injected at any time-point, which can be viewed as the bound *μ*. Furthermore, the input matrix, *B*, determines how much each variable is affected by the input signal and hence cannot be neglected.

As U is typically not centred around 0, we divide the input effect into a drift term, corresponding to the effect of the centre of U (*u*_c_) and an uncertainty term representing our lack of knowledge about the exact input value, i.e. the maximal difference, *u*_d_, between the centre and possible values of U. Thus, the whole input set is taken into account as $U=[u_{c}-u_{d};u_{c}+u_{d}]$; for instance, in the example above, *u*_c_=*μ*/2 and *u*_d_=*μ*/2. The drift and uncertainty terms are calculated using equation (3.3), and the bloating set will be the zonotope

3.6 $$\beta_{\mu }=(\psi (A,\tau )Bu_{c};\psi (A,\tau )Bu_{d}).$$

This calculation in this exact form is only possible if *A* is invertible—in the singular case we can use an approximation of equation (3.3), based on the integral of the Taylor-series of e^*As*^ and compute a bloating factor *β*_*ν*_ to correct for the small error thus introduced [20]:

$$\psi (A,t)=\sum_{i=0}^{\nu }\frac{A^{i}t^{i+1}}{(i+1)!}⊕\underset{\beta_{\nu }}{\underbrace{\left(0;e^{|A|t}-\sum_{i=0}^{\nu }\frac{|A{|}^{i}t^{i}}{i!}\right)}}$$

### 3.2. Parameter uncertainty

Often we have to make predictions but only have approximate values of the parameters contributing to matrix *A*, either due to imperfect knowledge of reaction rates [41], or because we can only control a reaction through some interaction that is not or cannot be modelled explicitly. Therefore, we derive a way to account for uncertainty in case the matrix *A* is also drawn from a set or ensemble of matrices. For example, we can consider the case where a single reaction rate, *k*, possibly affecting more than one element of *A*, is not known precisely or controlled. We assume that *k* has some plausible upper and lower bounds and hence it can be considered as coming from an interval centred at the nominal value $\hat{k}$, i.e. $k\in [\hat{k}-\delta ,\hat{k}+\delta ]$. We approach the problem by following the nominal dynamics using the previously defined matrix, *A*, and defining a bloating term to enclose all solutions arising from admissible parameter values, so that

$$R_{i+1}(A(k))\subseteq R_{i+1}(A(\hat{k}))⊕\beta_{\delta },$$

where *A*(*k*) denotes the matrix formed using the parameter value *k*. For positive *x*, we can bound *A*(*k*)*x* with $A(\hat{k})x+Dx$, where *D* is the *n*×*n* matrix computed as $|A(\hat{k}+\delta )-A(\hat{k})|$. To make *Dx* independent of the state variable—and thus derive a general formula—we use a conservative estimation of *x*: $Dx\leq D|x{|}_{\max}$, where $|x{|}_{\max}$ can be approximated generally for the whole algorithm, or, more practically, in each time-step based on the states in the current reachable set. We derive $|x{|}_{\max}$ from the zonotope definition in equation (2.1): given a reachable set in the form (*c*,*G*), the *i*th coordinate of the maximal vector is computed by the formula

3.7 $${\left\{|x{|}_{\max}\right\}}_{i}={\left\{c\right\}}_{i}+\sum_{k=1}^{p}|{\left\{g_{k}\right\}}_{i}|.$$

Note that $|x{|}_{\max}$ might not be in the reachable set, but there is at least one point for each coordinate for which ${\left\{x\right\}}_{i}={\left\{|x{|}_{\max}\right\}}_{i}$ holds (see electronic supplementary material, figure S2(b)). $|x{|}_{\max}$ is derived as |c|+∑|G| with all summations carried out *by rows*. We also take into account a rough estimate of the next reachable set, so that in non-converging cases $|x{|}_{\max}$ is not underestimated: $|x{|}_{\max}=\max\{|c|+\sum |G|,\;e^{A\tau }(|c|+\sum |G|)\}$.

For each coordinate of vector $D|x{|}_{\max}$ the difference between dynamics under any *k* and the centre, $\hat{k}$, can be enclosed in the zero-centred set,

3.8 $$\forall d_{i}={\left\{A(k)x\right\}}_{i}-{\left\{A(\hat{k})x\right\}}_{i};\;d_{i}\in (0;{\left\{D|x{|}_{\max}\right\}}_{i}),$$

from which a zonotope considering all coordinates can be obtained as $\left(0;\text{diag}\{D|x{|}_{\max}\}\right)$. From here, we proceed considering this zonotope as an input set centred at 0—in agreement with the fact that trajectories of the nominal value, $\hat{k}$, are already calculated through the transition matrix. Therefore, the bloating set accounting for parameter uncertainty, *β*_*δ*_, can be computed as

3.9 $$\beta_{\delta }=(\underline{0};\psi (A,\tau )\text{diag}\{D|x{|}_{\max}\}).$$

### 3.3. Nonlinear systems

Biological systems are often nonlinear, as even the simplest dimer-formation requires second-order rate laws that cannot be eliminated from the system. Nonlinear systems can lead to complex and unpredictable behaviour and their analysis can be difficult. The most popular way to overcome this is by performing a (piece-wise) linearization of the system. Here, we adopt this strategy, and at each step we linearize the system around its current centre using a first-order Taylor expansion, as described in [42]. This results in a system which is piece-wise linear, and has LTI properties in any interval, but which is overall time-varying; of course, this is only an approximation of the real underlying dynamics. The linearized system calculated at time *iτ* is

3.10 $$\dot{x}(t)={\frac{\partial f}{\partial x}|}_{x=c_{i}}(x(t)-c_{i})+f(c_{i})=A_{i}x(t)-A_{i}c_{i}+f(c_{i}).$$

This system can be used to obtain the next reachable set together with a bloating factor that accounts for the difference between the original and the linearized dynamics

3.11 $$R_{i+1}=(c_{i}+\psi (A_{i},\tau )f(c_{i});\;e^{A_{i}\tau }G_{i})⊕\beta_{\epsilon },$$

where *β*_*ϵ*_ can be computed iteratively as proposed in [42]. Here, we use the zonotopic set representation to obtain a tight estimate of the effect of nonlinearity.

Consider the error function *ϵ*(*x*)=|*f*(*x*)−[*A*_*i*_(*x*−*c*_*i*_)+*f*(*c*_*i*_)]|—using a substitution of *x* by (*c*_*i*_+*dx*), we obtain *ϵ* as a function of *dx*, the distance between a point and the centre. We define an upper bound for *dx*, as in equation (3.7): ${\left\{|dx{|}_{\max}\right\}}_{i}=\sum_{k=1}^{p}|{\left\{g_{k}\right\}}_{i}|$, and approximate *ϵ*(*x*) with a function monotonously increasing with *dx*. Hence, we obtain an estimation for which $\epsilon (x)\leq \hat{\epsilon }(|dx{|}_{\max})$ and construct a generator set

3.12 $$G_{l}=\text{diag}\{\hat{\epsilon }(|dx{|}_{\max})\},$$

which represents a bound on the nonlinearity of the system.

To obtain the reach set of the time-interval [*iτ*,(*i*+1)*τ*] further approximations have to be applied to enclose all trajectories between the two time-points. As the piece-wise linear system is time-varying, the estimation of the time-interval [0,*τ*] is not sufficient. Instead, as mentioned before (see also in electronic supplementary material, figure S1(b)), the convex hull of two delimiting reach sets is calculated and bloated, the bloating factor for which can be derived in several ways, e.g. as in [9] or [42].

Although the computation in equation (3.11) assumes a constant time-step, it can be modified to incorporate variable steps, *τ*_*i*_, without loss of efficiency, as in the nonlinear case the matrix exponential, e^*A*_*i*_*τ*^, should anyway be computed in each iteration. Consequently, the first-order linearization used above can be combined with a higher-order, adaptive method by computing the central trajectory (*c*_*i*_) and each time-step (*τ*_*i*_) with the higher-order numerical technique, followed by reachable set computation based on these quantities. This way stiff systems with rapidly changing dynamics can be tackled without significant additional computing time, and slowly changing systems can gain a speed-up compared with fixed time-step calculations. Alternatively, we could replace the linearization with a more flexible description of the stochasticity than the LNA, such as moment closures [30] or finite-state projection methods [43].

## 4. Example applications

### 4.1. Control of gene expression noise

The first example considered is a controlled stochastic gene expression system [28,44]. In spite of its simplicity, this model is one of the most important examples in practice as protein production is a necessary and elementary building module in real and synthetic biological systems. Many applications might rely on the steady operation of such a unit, hence it is of great practical interest to know what the achievable noise levels and possible states of operation are. Let us take a simple depiction of protein production that focuses on two macromolecules, mRNA and protein, captured in two quantities of interest (mRNA and protein copy numbers, *m* and *p*, respectively). The overall process is broken up into four reactions, as follows:

$$\begin{array}{rl} \emptyset & \overset{k_{1}\;\mu }{\to }\text{mRNA}(\text{transcription}),\\ \text{mRNA} & \overset{k_{2}}{\to }\emptyset \;(\text{mRNA degradation}),\\ \text{mRNA} & \overset{k_{3}}{\to }\text{mRNA}+\text{protein}\;(\text{translation})\text{and}\\ \text{protein} & \overset{k_{4}}{\to }\emptyset \;(\text{protein degradation}), \end{array}$$

where the symbol ∅ represents that a molecule is produced from (or degraded into) some substance not modelled in the representation. This system translates into the stoichiometry matrix

$$S=\left[\begin{array}{cccc} 1 & -1 & 0 & 0\\ 0 & 0 & 1 & -1 \end{array}\right],$$

with each column standing for molecular abundance changes during a reaction of the ones listed above. The propensity of each reaction is formed by its characteristic rate and the molecular species’ abundances,

$$a_{1}=k_{1}\mu ;\;a_{2}=k_{2}m;\;a_{3}=k_{3}m\;\mathrm{and}\;a_{4}=k_{4}p.$$

In this example, the dimensionless multiplier in the first reaction, *μ*, represents our control over the transcription rate, assuming either discrete (*μ*=0 or *μ*=1) or continuous (*μ*∈[0,1]) values. A typical control signal would be a sequence of zeros and ones, delivered through, e.g. a light-sensitive set-up [45], switching transcription on and off for some time period. After performing the LNA, we obtain a set of five ordinary differential equations, determining the time-evolution of the mean of *m* and *p*, the variance of *m* (*σ*_*m*_), the covariance of the two species (*σ*_*mp*_) and finally the variance of the protein abundance (*σ*_*p*_),

$$\frac{d}{dt}\left[\begin{array}{c} m\\ p\\ \sigma_{m}\\ \sigma_{mp}\\ \sigma_{p} \end{array}\right]=\left[\begin{array}{c} k_{1}\mu -k_{2}m\\ k_{3}m-k_{4}p\\ -2k_{2}\sigma_{m}+k_{1}\mu +k_{2}m\\ k_{3}\sigma_{m}-(k_{2}+k_{4})\sigma_{mp}\\ 2k_{3}\sigma_{mp}-2k_{4}\sigma_{p}+k_{3}m+k_{4}p \end{array}\right].$$

The system is started from $I=\underline{0}$, corresponding to no previously produced target molecules in the system and we investigate the reachable sets up to time *T*=10 with time-step *τ*=0.01. The time-units are normalized according to the protein’s average turnover time, so that we gain a general model that might be later scaled to reflect a specific macromolecule. Therefore, *k*_1_ has units of [molecules/time-unit (t.u.)], and all other *k*_*i*_ are in [1/t.u.]. We choose the reaction rates $\underline{k}=[100,5,100,1]$ to show biologically relevant ratios of reaction speeds and steady states: under constitutive production there will be on average 20 mRNA and 2000 protein molecules.

**Figure 1.**
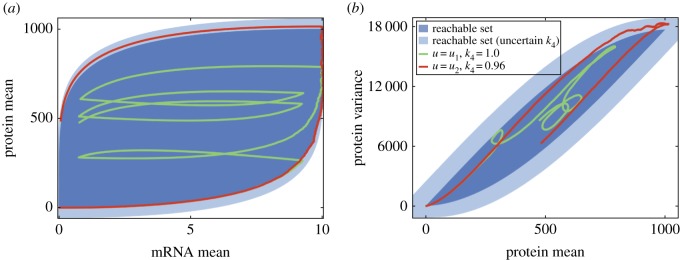
Reachable states of the stochastic gene expression system with controlled transcription and additional uncertainty. Blue-shaded regions show projection of the final reachable set to (*a*) the mRNA mean–protein mean plane and (*b*) the protein mean–protein variance plane. Dark and light blue shades indicate reachable sets without and with 5% uncertainty in parameter *k*_4_. Red and green example trajectories are calculated from 10 000 exact simulations, under the input sequences *u*_1_=[1,0,1,1,1,0,1,0,1,0] and *u*_2_=[1,1,1,1,1,1,1,1,0,0,0,0,0,1,0,1,1,0,1,1], respectively, with protein degradation values as indicated in legend.

Figure 1 shows projections of the final reachable set together with two sample trajectories. The samples are computed as the mean and variance of a population consisted of 10 000 direct realizations of the original stochastic system. The input signal *μ*(*t*) is defined as a piece-wise constant function with values randomly drawn from {0,1} and switching every 60 or 30 s (*u*_1_ and *u*_2_, respectively); the signal is kept the same for all realizations contributing to a particular trajectory in figure 1. We also take into account some uncertainty regarding the protein degradation rate, i.e. that the value of *k*_4_ is unknown or can be externally changed to values within 5% of the nominal value, 1. The light blue regions in figure 1 show the estimate of the reachable set under such uncertainty. Interestingly, while a substantial part of the mRNA—protein mean space can be covered, the reachable states on the protein mean—variance plane are limited to a very narrow band. Therefore, if relying on the production of a protein with this module we will have to make a compromise: either have a low number of proteins produced, or a high amount but with great variability (as may be expected given the Poisson nature of this process). In addition, we find that uncertainty in the degradation rates has a profound effect on the general uncertainty of both the mean and variance levels (see electronic supplementary material, figure S3)—in agreement with previous findings on the importance of degradation [46,47].

### 4.2. Model validation

**Figure 2.**
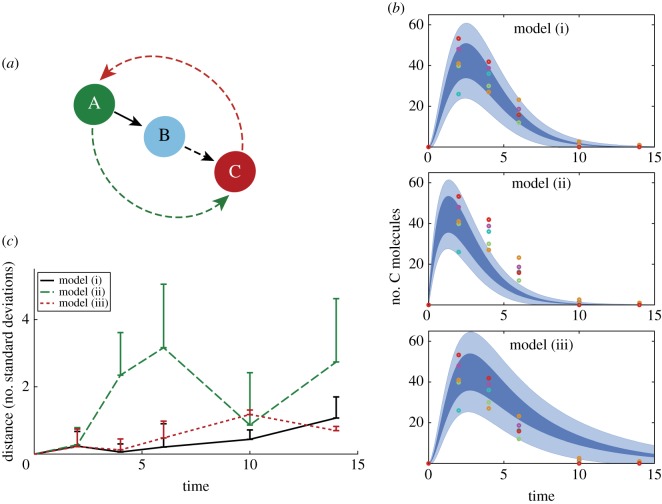
Model evaluation by comparing reachable sets and single measurements. (*a*) Schematics of the three reaction chain models. Models differ in rates corresponding to dashed arrows. (i) *k*_BC_=1, *k*_AC_=*k*_CA_=0. (ii) *k*_BC_=0.1, *k*_AC_= 0.9, *k*_CA_=0. (iii) *k*_BC_=1, *k*_AC_=0, *k*_CA_=0.2. (*b*) Reachable region over time of the output (molecule C) starting from the initial set 80≤*A*_0_≤120, *B*_0_=*C*_0_=0. Dark areas represent reachable values of the mean, light blue shades are the ± 1 s.d. region computed with the maximal reachable value of the variance. Coloured circles are sample points taken from single exact simulations of model (i). (*c*) Distance of observation points from the reachable set of mean values. Lines show the average distance of observed data points at each time of measurement, the top of error bars depict the maximal distance at the evaluation points (colours as indicated in legend). Distances for each model are normalized by the maximal reachable standard deviation value. Time in (*b*) and (*c*) is in arbitrary units based on the macromolecular production rate, such that with maximal interaction strength, on average one product molecule is created in 1 t.u.

In our second application, we consider a chain of three molecules affecting each others’ production and demonstrate how our reachability analysis can contribute to model validation [48] for stochastic systems. The molecules A, B and C in figure 2 can represent any chain of interacting species with similar reaction networks; for example, a simple model of transcription factors, where only protein levels are modelled explicitly. The regulatory effect of these molecules is through the production rate of their target species. Three different wiring schemes are considered: (i) molecule A induces molecule B, which in turn activates the production of C; (ii) molecule A also activates molecule C, such that this effect is more profound than the activation via B; (iii) molecule C feeds back onto, and induces the production of A. In each model, we simplify the mathematical description by assuming equal degradation rates for all species; hence the models can be summarized by five parameters: *k*_AB_, *k*_BC_, *k*_AC_, *k*_CA_ and *k*_deg_, where the subscript *XY* refers to the activation of *Y* by *X*. In all models *k*_AB_=1 and *k*_deg_=0.8, and the other parameters are chosen to reflect the connections of the model and produce similar maximal values in the output, C. All reaction rate values have units of [1/t.u.], where the arbitrary unit of time relates to the speed of changes in the system. However, the general shape of dynamics is determined by the relative weights of certain reactions, which the above simplified rate values show more clearly. These can be later scaled to represent a particular system with known time-scales. For example, if it is known that the production of molecule B through the activation of A results in approximately 500 molecules each hour, *k*_AB_ and *k*_deg_ should become 500 and 400 [1/hour], but the following analysis would only differ in the scale of the horizontal axis.

In order to model measurements, we generate five individual stochastic trajectories from model (i), with parameter values *k*_BC_=1, *k*_AC_=*k*_CA_=0 and an initial A value randomly chosen from the range [80,120]. We then sample the amount of molecule C, associated with the experimentally measurable output, from these simulations at six time-points; and evaluate the distance of each measurement point from the reachable set of mean values at that specific time. The distance is normalized by the maximum reachable value of standard deviation and consequently zero for all points within the reachable set. As figure 2*b*,*c* show, it is very unlikely that the observations arose from model (ii). Although the true model shows the best correspondence to the data, model (iii)—representing a feedback system—cannot be discarded due to the wide range of values molecule C can reach in that specific wiring scheme. If the relative noise level is reduced by raising the average initial abundance (to approx. 300 molecules), or measurements from another species (e.g. molecule A) is also available, the distinction between different models becomes clear with model (i) unambiguously fitting the data best.

This should only serve as an illustration of the potential use of reachability analysis for model (in)validation. Coupled with, e.g. experimental design methods [49,50], we can, for example, iteratively rule out candidate models.

### 4.3. Bistable system

Our last example is a model exhibiting bistability, on which we demonstrate limitations of zonotope-based reach set computation, but also how reachability analysis can be used for *qualitative* exploration of biological system dynamics. As bistability plays a crucial role in cellular decision making [51], we choose a concrete stem cell differentiation model to examine this phenomenon. The model describes the interaction of the Oct4-Sox2 complex with stem cell marker Nanog [52]. Although it is a simplified representation of the underlying network that summarizes numerous reactions into rates used in modelling, it still produces bistable behaviour. The model has two variables (*N* and OS) and five reactions,

$$\begin{array}{rl} a_{1} & =k_{1}\cdot {\left(\frac{k_{2}{\mathrm{OS}}^{\gamma_{\mathrm{os}}}}{(K+{\mathrm{OS}}^{\gamma_{\mathrm{os}}})k_{\deg}}\right)}^{2};\;a_{2}=k_{\deg}\mathrm{OS};\\ a_{3} & =\frac{k_{3}{\mathrm{OS}}^{\gamma_{\mathrm{os}}}}{K+{\mathrm{OS}}^{\gamma_{\mathrm{os}}}};\;a_{4}=\frac{k_{4}N^{\gamma_{n}}}{K+N^{\gamma_{n}}}\;\mathrm{and}\;a_{5}=k_{\deg}N, \end{array}$$

where the reactions stand for auto-activated production of the Oct4-Sox2 complex, degradation of the complex, Oct4-Sox2 activated production of Nanog, auto-activated production and degradation of Nanog, respectively. Accordingly, the stoichiometry matrix of the system is

$$S=\left[\begin{array}{ccccc} 1 & -1 & 0 & 0 & 0\\ 0 & 0 & 1 & 1 & -1 \end{array}\right].$$

We take a set of parameter values from [52] that makes the model bistable,

$$[k_{1},k_{2},k_{3},k_{4},k_{\deg},K,\gamma_{\mathrm{OS}},\gamma_{N}]=[0.03,50,0.1,14,1,10,1,2].$$

Here, we focus on how bistability affects our prediction of the average behaviour. Therefore, in practice, our analysis is equivalent to computing the reachable sets of the corresponding deterministic system. We fix rate parameters to the above values, and introduce uncertainty through allowing a range of initial values. As noise in the initial Oct4-Sox2 concentration, OS_0_, has no significant effect on the dynamics, we fix its value to 60. Similarly, variance and covariance values do not influence mean Nanog level, hence their value is also without variability, *σ*_os_=10, *σ*_os−*n*_=1, *σ*_*n*_=0.1. On the other hand, as the system is in a bistable regime, Nanog initial values determine which of the two fixed points—corresponding to high-Nanog, stem cell phenotype or low-Nanog, differentiated phenotype—is reached by the system. Therefore, we explore different initial values of Nanog, *N*_0_, with a 25% uncertainty in each case.

**Figure 3.**
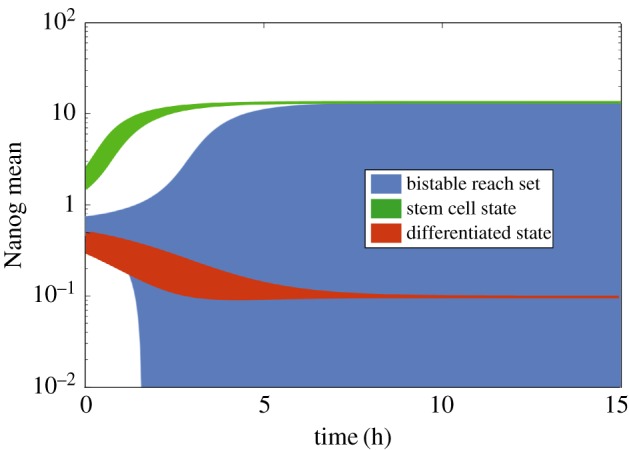
Investigating bistability via reachable sets of a stem cell differentiation model. Shaded regions show a conservative estimate of the achievable mean Nanog levels over time. The dark blue region is an over-approximation of the reachable set of the bistable system, when the analysis is started from an initial set of bistability, i.e. a set enclosing states leading to both fixed points. *N*_0_=0.6±0.15. Red and green shades show reachable sets computed from ‘monostable’ initial sets, from which all trajectories converge to the differentiation fixed point (low-Nanog level, in red) or to the stem cell fixed point (high-Nanog level, in green). Initial Nanog values are 0.4±0.1 and 2±0.5, respectively.

The value 0.6 is close to the boundary between the two basins of attraction (c.f. fig. 2A in [52]). Hence, the initial set I=[0.45,0.75] inevitably contains a sample of states from both basins. We first compute the reachable set up until 15 arbitrary time units. As zonotopes are limited to describe centrally symmetric and convex reach sets, the set enclosing both high- and low-Nanog steady states also includes a set of implausible, negative values that arise as the symmetric counterparts of the highly positive (greater than 10) Nanog values. We exclude this range of values as it is clearly an artefact of set representation and concentrate on the strictly positive subset of reachable states. Figure 3 shows Nanog mean values in blue on a logarithmic scale as a function of time. As a comparison, we also conduct reachability analysis restricted to the stem cell or differentiated cell regime. The former is started from the initial set I=[1.5,2.5] and the latter from Nanog values *N*_0_∈[0.3,0.5]. The collective evolution of trajectories is shown with red and green reach sets in figure 3.

It is clear from the comparison of the shaded regions in figure 3 that the final reachable set obtained with *N*_0_=0.6 indeed contains both steady states. Furthermore, the upper limit of the reach set is not only conservative, but also tight. Therefore, the only crude over-approximation is due to symmetry and the high-Nanog state is well outlined even in this analysis. In addition, obtaining negative reachable sets for variables that should not assume negative values can be used as an indicator of discontinuous behaviour within the analysis. After such an observation, restricting the set of initial values—similarly to how the second and third reachable sets are obtained in figure 3—can shed light on the underlying dynamics.

## 5. Discussion

In this work we have introduced a method to compute the states that a stochastic biochemical network can access under a control input signal. The method is particularly applicable to determine reachable mean–variance values of the investigated species, and hence estimate noise levels of a system. Our aim is to provide a computationally efficient tool that could be used in the search for the best modelling description or regulatory design of stochastic networks.

Here, we used the linear noise and moment expansion approximations to extract deterministic equations of the mean and covariance matrix that served as a basis for our reachable set computation. Both can be accessed on GitHub as parts of our Python package, MEANS [31]. We have presented a general algorithm for linear and nonlinear reachability analysis based on zonotopes, together with formulae for handling relevant input types: addition of molecules and control of a reaction rate. All steps are implemented in Matlab to semi-automatically generate the reachable sets for a problem defined in terms of a transition function, input matrix and bounds, and parameter uncertainty. The algorithm can be executed on any given set of ODEs describing some characteristics of the system, hence it is applicable regardless of the approximation method used for the generation of equations and also to deterministic biological models.

We demonstrate the method on three schematic models of biological macromolecules: a controlled gene expression system; a cascade of modifying molecules, e.g. transcription factors; and a bistable model of stem cell differentiation. In all examples the approximation is conservative, hence trajectories randomly generated from a set of admissible signals and initial values are confined within the set our method predicts. In linear cases, if we have precise knowledge of the parameter values and input bounds, the predicted reachable set will be exact as well, i.e. each point in it can be actually accessed by an appropriate input sequence. This cannot be ensured for nonlinear systems (or uncertain rate values) as the conservative estimates used for the nonlinearity (or reaction rate effect) are obtained from approximations the system might not take. However, our estimation reflects the general characteristics of the system—e.g. if some variables of a generally nonlinear system have linear equations—and hence unnecessary over-approximations are avoided to provide a tight estimate. This is crucial in the evaluation of various control designs on the basis of mean–variance values reachable through them.

Our method is based on a zonotope representation of reachable, initial and parameter sets. Set operations can be efficiently computed using this representation method, which allows us to apply our reachability analysis to many-variable, complicated biological networks. However, the number of generator vectors in the reachable set increases in every iteration, and for a long time horizon or small time-step the algorithm can become expensive regarding memory space. In smaller systems, such as our examples, this effect is still negligible, but for high-dimensional cases the increase is more significant. For such cases one can turn to the zonotope-reduction technique presented by Girard [9] to limit the size of zonotopes to an adjustable value.

Note also that zonotopes, and hence the sets in our analysis, are by definition convex and centrally symmetric. Therefore, systems where the actual reachable set is concave or consisted of multiple sets—especially networks with bi- and multi-stability—will be largely over-approximated. In our last example, we have tested this aspect of the algorithm on a differentiation model with bistable dynamics. As the two expression level states in such cases are typically of different magnitudes, our method is forced to enclose a high proportion of negative values besides also incorporating a non-accessible region between the two states. However, we also found that this drop in approximation quality is a good indicator of the system entering a bistable regime of initial conditions/input signal. A similar over-approximation case can arise for imprecise parameter values of the transition matrix: although admissible parameter sets are symmetric sets given as a nominal value with error bounds, the influence of parameters is usually not symmetric on the reachable set. Therefore zonotopes lead to a rough approximation when some rates show high uncertainty. Just like in the case of multi-stability, a preliminary analysis using our general method can reveal these issues, and point one towards a more exhaustive study using a series of reachable sets for fixed values (or small intervals) of initial values or rate parameters to cover the range of interest and extrapolate for tighter approximation.

As often the case in the analysis of biochemical systems, incomplete information about the model structure and crucial constants of the system makes a detailed and informative analysis impossible. Our method is not applicable for the exploration of differences in model structures; this problem can be treated implicitly by either doing independent analysis of all candidate models or choosing rate parameter uncertainty such that zero (i.e. no interaction) is among the admissible values. The latter is likely to give rise to an over-generalized approximation, as described above; while the first one is only advisable for a small number of possible models, like in our second example. Furthermore, limited measurements and high levels of noise can also influence the method’s power for validation, as figure 2*c* demonstrates: in such cases flexible models might be favoured even over the true model. Therefore, we advise the use of other tools, such as topological sensitivity analysis [53] coupled with our method for a more thorough investigation of model space.

## Supplementary Material

Supplementary Derivations and Results

## Data accessibility

This article has no additional data.
