# Supplementary material for: Control mechanisms for stochastic biochemical systems via computation of reachable sets
Source: R Soc Open Sci. 2017 Aug 23;4(8):160790. doi: 10.1098/rsos.160790 (PMC5579072; doi:10.1098/rsos.160790)
Supplement: Supplementary Derivations and Results [file rsos160790supp1.pdf]

Supplementary information for *Control mechanisms for stochastic biochemical systems via computation of reachable sets*

Eszter Lakatos and Michael P.H. Stumpf

Illustration of zonotope generation and the general algorithm for computing a time-interval reach set

Figure S1(a) shows how the generator set of a zonotope is added up in the Minkowski sense to create the zonotope.

Figure S1(b) provides an illustration of the most general algorithm for computing the reachable set for a time interval, $[t, t+\tau]$. However, as discussed in section 3, an approximation of this method is preferred if the problem permits, since the convex hull of two zonotopes (such as on the third and final panel of the figure) is no longer a zonotope. This leads to the loss of computational efficiency achieved in zonotope representation.

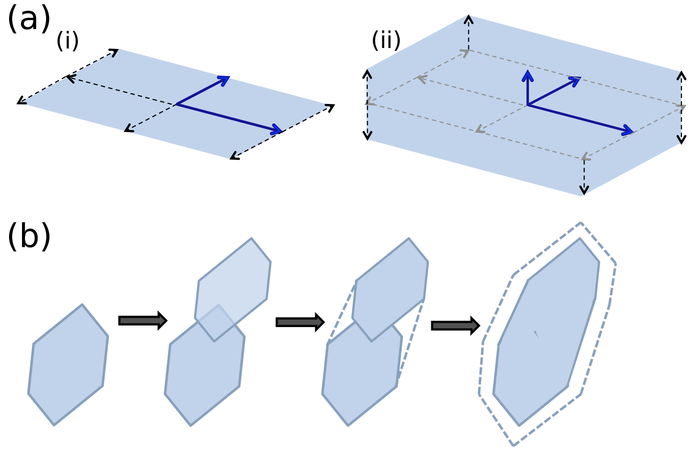

Figure S1: (a) Example of a zonotope (light blue shaded area) with two generators (blue arrows) **(i)**. **(ii)** The same zonotope after the addition of a third generator. **(b)** General method for the computation of reachable set for a time interval. Given the reachable set at time t (shaded area in leftmost panel), it is propagated into the reachable set at $t + \tau$ (second from the left), then the convex hull of the two sets are computed (third panel) and finally this set is enlarged to account for trajectories of non-linear dynamics (area encircled by dashes in rightmost panel).

Illustration for the computational steps in reachable set calculation

Figure S2 provides a visual demonstration of the major computational steps introduced in the main text. The toy model used for illustration is the *deterministic* system with transition matrix $A = \begin{bmatrix} -3 & 0 \\ 2 & -1 \end{bmatrix}$ that can be interpreted as a biological system with the following reactions

$$x_1 \xrightarrow{3} \emptyset; \quad x_1 \xrightarrow{2} x_1 + x_2; \quad x_2 \xrightarrow{1} \emptyset.$$

In addition, there is a fourth reaction $\emptyset \xrightarrow{u} x_1$ under our control, providing controllable input to the system. We assume our knowledge of the starting state is inaccurate and given with error bounds: $x_1(0) = 20 \pm 5$ and $x_2(0) = 35 \pm 10$. In our zonotope formalism the initial set is captured as $\mathcal{I} = \left(\begin{bmatrix} 20 \\ 35 \end{bmatrix} ; \begin{bmatrix} 5 & 0 \\ 0 & 10 \end{bmatrix} \right)$. Figure S2(a) shows the first few reachable sets computed as this initial set is propagated through the reachability algorithm, without any external input. Note that, as the transition matrix defines a stable, contracting system, the reachable sets get smaller and show convergence towards the origin, the stable steady state of the system.

Figure S2(b) illustrates the computation of quantities $|\mathbf{x}|_{max}$ and $|\Delta x|_{max}$ – used in the correction terms for parameter uncertainty and non-linearity – on a two-dimensional zonotope. The illustration also highlights that the actual state corresponding to the coordinates $|\mathbf{x}|_{max}$ or distance $|\Delta x|_{max}$ is usually not part of the zonotope.

Finally, the bloating used in the propagation of systems under input is demonstrated in Figure S2(c). Given the reach set, $\mathcal{R}_1$, and an input signal that might assume any values between 0 and 100, we first compute the reachable set with the fixed value $u = 50$. In the next step the set is bloated with a set of generators such that the two extrema (0 and 100) are both accounted for; giving rise to a reachable set, $\mathcal{R}_2$, containing all states achievable.

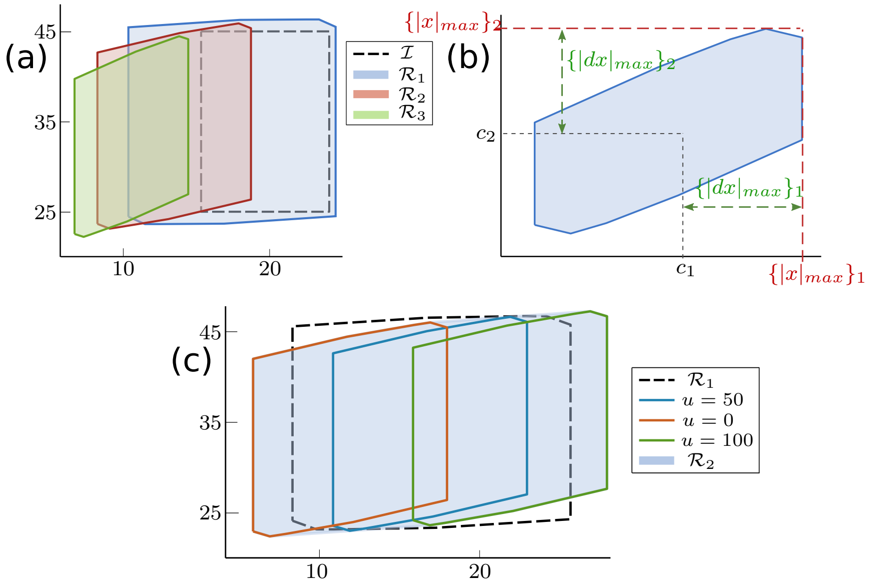

Figure S2: Examples of reachable set computation. **(a)** Propagation of the initial set, $\mathcal{I}$ (rectangle outlined by black dashes), following the system dynamics by transition matrix A, with zero input and time-step $\tau = 0.08$. Blue, red and green shaded areas show the set of states reachable in the time-intervals $[0, \tau]$, $[\tau, 2\tau]$, and $[2\tau, 3\tau]$, respectively. **(b)** Two-dimensional example of the computation of $|\mathbf{x}|_{max}$ and $|\Delta x|_{max}$. The centre of the zonotope and its respective coordinates are indicated with black dotted line, maximal coordinates with red dashes. Green dashes indicate the distance defining $|\Delta x|_{max}$. **(c)** Computation of bloating set accounting for a range of input signal values. Black dashes show the original reachable set, blue solid line the reachable set obtained with the middle of input range, 50. Blue shading represents the bloated reachable set. Dark orange and green shapes outline reachable sets with 0 and 100 input, respectively, which are both contained in the bloated set.

Analysis of the controlled gene expression system with uncertainty in all parameters

We also studied the set of states reachable in the controlled gene expression system, in case both degradation rates (k_2 and k_4) or all four parameter values (k_1, k_2, k_3, k_4) have 5% uncertainty compared to their nominal value. The reachable sets are shown in Figure S3 together with the previously obtained reach sets from Fig. 1(b). Interestingly, the two degradation rates contribute a dominant proportion to overall uncertainty (as described by the extension of the reachable set). Besides, uncertainty in rates of mRNA dynamics have a strong effect on the covered space (since any fluctuation in mRNA levels is amplified by translation), hence the major contribution of k_2 as shown by the third (purple) reach set.

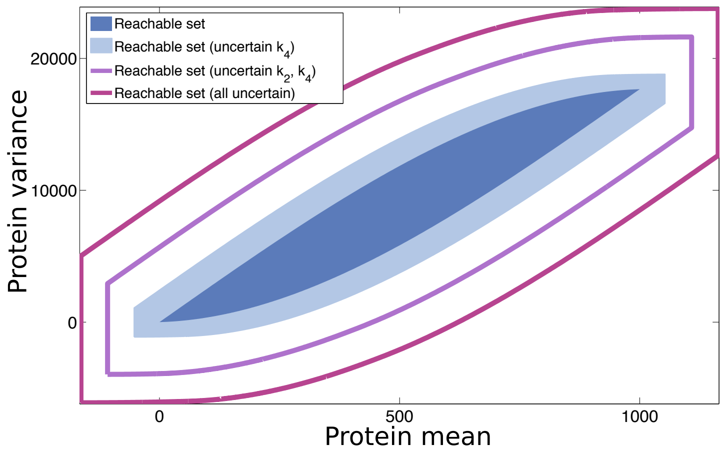

Figure S3: Reachable states of the stochastic gene expression system with controlled transcription and additional uncertainty. Light and dark blue shades represent reachable sets identical to those shown in Figure 1(b) (reach set computed with fixed k_4 and k_4 with 5% uncertainty). The area inside the purple and magenta limits show the sets reachable if both degradation rates or all 4 parameter values are subject to 5% uncertainty, respectively.
